# Three-Year Successful Cinacalcet Treatment of Secondary Hyperparathyroidism in a Patient with X-Linked Dominant Hypophosphatemic Rickets: A Case Report

**DOI:** 10.1155/2014/479641

**Published:** 2014-02-10

**Authors:** Diana Grove-Laugesen, Lars Rejnmark

**Affiliations:** Aarhus University Hospital, Department of Endocrinology and Internal Medicine, Tage-Hansens Gade, 8000 Aarhus C, Denmark

## Abstract

Hypophosphatemic rickets (HR) is a rare inherited disorder characterized by a classic rickets phenotype with low plasma phosphate levels and resistance to treatment with vitamin D. Development of secondary hyperparathyroidism (SHPT) as a direct consequence of treatment is a frequent complication and a major clinical challenge, as this may increase risk of further comorbidity. Cinacalcet, a calcimimetic agent that reduces the secretion of PTH from the parathyroid glands, has been suggested as adjuvant treatment to SHPT in patients with HR. However, only two papers have previously been published and no data are available on effects of treatment for more than six months. We now report a case of 3-year treatment with cinacalcet in a patient with HR complicated by SHPT. A 53-year-old woman with genetically confirmed X-linked dominant hypophosphatemic rickets developed SHPT after 25 years of conventional treatment with alfacalcidol and phosphate supplements. Cinacalcet was added to her treatment, causing a sustained normalization of PTH. Ionized calcium decreased, requiring reduction of cinacalcet, though asymptomatical. Level of phosphate was unchanged, but alkaline phosphatase increased in response to treatment. Cinacalcet appeared to be efficient, safe, and well tolerated. We recommend close control of plasma calcium to avoid hypocalcemia.

## 1. Introduction

Familial hypophosphatemic rickets (HR) is a rare inherited disorder characterized by renal phosphate wasting and inappropriately low levels of calcitriol causing hypophosphatemia and abnormal bone mineralization, first described by Albright [1]. The typical phenotype is characterized by severe bone deformities and growth retardation presenting in childhood with bowing of legs and shortness of length. In adulthood, the disease is often complicated by osteomalacia with bone pain and arthralgias due to joint deformities and enthesopathies. Occurrence of dental problems like tooth abscesses is also common [2, 3].

In most instances, HR is caused by mutations in genes affecting the metabolism of fibroblast growth factor 23 (FGF-23) [4]. The prevailing form of HR is X-linked dominant hypophosphatemia (XLH) caused by an inactivating mutation of the PHEX gene [5].

Medical treatment of HR includes activated vitamin D analogues and supplements with high amounts of oral inorganic phosphate salts which heal osteomalacia, but neither corrects the biochemical abnormalities nor the bone deformities [6]. The renal phosphate leak is not improved; in contrary, treatment has been shown to increase levels of FGF23 [7]. Also, phosphate supplements tend to increase PTH levels [8] and secondary or even tertiary hyperparathyroidism may develop in response to treatment [9]. Secondary hyperparathyroidism (SHPT), reported in as many as 25% of treated patients, is associated with an increased risk of nephrocalcinosis and hypertension and may require cessation of treatment [10].

Cinacalcet is a calcimimetic agent which increases the sensitivity of the calcium-sensing receptor (CaSR) to extracellular calcium, causing reduced release of PTH [11, 12]. In the setting of chronic kidney disease, long term treatment with cinacalcet has proven efficient in controlling biochemical parameters associated with SHPT [13]. A study of the acute effect of cinacalcet in XLH demonstrated reduced levels of PTH and increased renal phosphate reabsorption [14]. However, no long term data on the effects of cinacalcet on calcium-phosphate homeostasis in XLH patients with SHPT are available. Here, we report the outcome of three-year treatment with cinacalcet in a patient with XLH and SHPT.

## 2. Case Report

A white Caucasian woman, born in 1954, developed SHPT at the age of 53 approximately 25 years after onset of treatment of XLH. As a child, the patient was in 1957 referred for bowing of lower legs, interpreted as a physiological phenomenon that would correct itself. At the age of 14, the patient was diagnosed with osteomalacia and started treatment with cholecalciferol. At the age of 22 the patient was osteotomized in both tibiae and a diagnosis of familial hypophosphatemic rickets was suspected. The patient was referred to a medical department where she presented with shortness of stature, discrete bowing of legs, and increased lumbar lordosis. She had a history of tooth abscesses and complained of patellar subluxations and knee and hip arthralgias. X-ray demonstrated early signs of osteoarthritis in knees and hips. The diagnosis was established in 1977 based on biochemical and urine analysis showing a reduced renal tubular maximum reabsorption rate of phosphate (TmP/GFR 0.48 mmol/L: reference interval 0.6–1.4 mmol/L), with no hypercalciuria (urinary calcium 2.9 mmol/d: reference interval 1.2–3.8 mmol/d) but low plasma levels of calcitriol (16 pmol/L: reference interval 24–158 pmol/L) in the presence of hypophosphatemia (plasma phosphate 0.6 mmol/L: reference interval: 0.8–1.5 mmol/L). An iliac crest bone biopsy revealed severe osteomalacia with low bone turnover. Due to low adherence to clinical follow-up visits, medical treatment with phosphate supplements and cholecalciferol was not initiated until five years later when the patient was 27 years old. Cholecalciferol was exchanged for an activated vitamin D analog (alfacalcidol) two years later. Repeated bone biopsy after two years of treatment revealed healing of mineralization defect, but complaints of joint ache endured and increased, necessitating knee and hip replacement in 2010 and 2012, respectively. At the age of 51 the patient was entitled incapacity benefit. Genetic analysis identified a missense-mutation in the PHEX-gene showing the patient to be heterozygous for c.1601 C>T, exon 15. The patient's sister and two children, a boy and a girl, share the same mutation.

**Figure 1:**
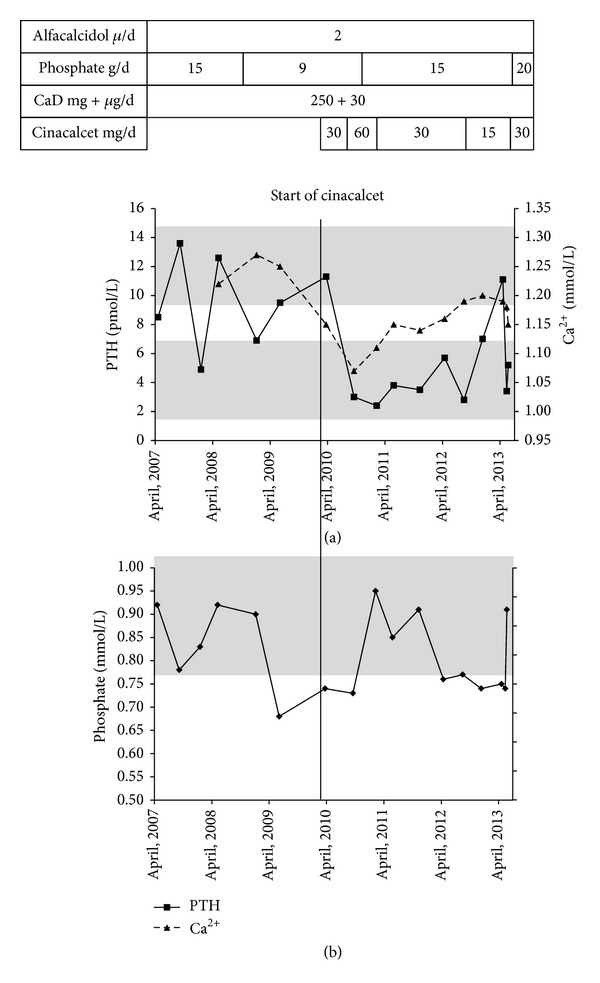
Plasma levels of PTH, ionized-calcium (Figure 1(a)), and phosphate (Figure 1(b)) three years before initiation of cinacalcet treatment and during the three years of cinacalcet treatment. Start of cinacalcet treatment, as indicated by the vertical line, was March 2010. Top bars show dosage of medical treatment. CaD; calcium carbonate 250 mg with 30 *μ*g/d (1200 IU) of cholecalciferol. Reference intervals of plasma PTH (1.6–6.9 pmol/L) are indicated by the lower grey area and plasma Ca^2+^ (1.18–1.32 mmol/L) by the upper grey area. Measurements of ionized calcium (Ca^2+^) were not available prior to May 2008 (Figure 1(a)). The reference interval of plasma phosphate was 0.76–1.41 mmol/L, as indicated by the grey area (Figure 1(b)).

**Figure 2:**
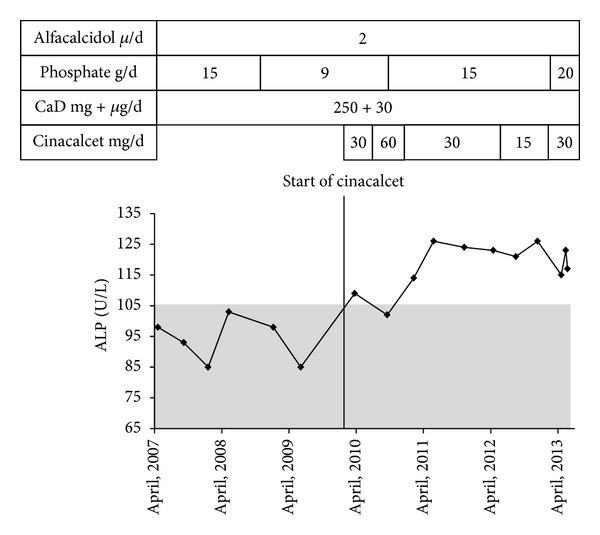
Plasma levels of alkaline phosphatase three years before initiation of cinacalcet treatment and during the three years of cinacalcet treatment. Start of cinacalcet treatment, as indicated by the vertical line, was March 2010. Top bars show dosage of medical treatment. CaD; calcium carbonate 250 mg with 30 *μ*g/d (1200 IU) of cholecalciferol. The reference interval of plasma alkaline phosphatase was 35–105 U/L as indicated by the grey area.

In 2007 the patient was diagnosed with SHPT which was not corrected by adjustment of therapy. Being severely symptomatic, continued treatment was required, and in March 2010 the patient consented to experimental treatment with cinacalcet in an attempt for curing her SHPT. Thirty mg of cinacalcet was added to her treatment as displayed in Figure 1. An attempt at increasing dosage to 60 mg was abandoned because of a fall in plasma calcium (October 2010, Figure 1(a)). Hereafter, PTH was stable and within the reference interval until the dosage of cinacalcet was reduced prior to planned hip surgery (30 mg every second day, August 2012) resulting in elevated PTH levels that endured until dosage of cinacalcet was adjusted at 30 mg per day (May 2013). Plasma phosphate was on average unchanged after addition of cinacalcet (Figure 1(b)). Plasma calcium was lower than prior to cinacalcet treatment but the patient did not experience hypocalcemic symptoms, and treatment was well tolerated without complaints of adverse effects (see Table 1). Following start of treatment with cinacalcet, plasma levels of alkaline phosphatase (ALP) rose significantly but stabilized (Figure 2).

## 3. Discussion

### 3.1. Effect on PTH and Calcium

To the best of our knowledge, this is the first report on long term use of cinacalcet as a therapeutic approach to SHPT in a patient with XLH. During the three years of treatment, PTH levels showed a sustained dose-dependent response to the treatment with cinacalcet and were maintained within the reference interval when dosage of cinacalcet was 30 mg per day or more. This is in accordance with studies on the effect of cinacalcet in patients with primary hyperparathyroidism [15], and observations in chronic kidney disease where cinacalcet has proven efficient in the long term control of SHPT [13].

Previously, only one case report has described the use of cinacalcet in a patient with XLH complicated by SHPT, as Yavropoulou et al. [16] reported successful treatment with cinacalcet 60 mg a day for a period of six months with sustained normalization of PTH. Total calcium levels were normal at all times with a tendency to decline, although ionized calcium levels surprisingly tended to rise. In contrast, our data suggest that cinacalcet lowers plasma calcium levels in patients with HR, similar to the effect reported in patients with primary hyperparathyroidism [15].

We found only one clinical study on the effect of cinacalcet in patients with XLH. Alon et al. [14] studied eight children with XLH but with normal plasma levels of ionized calcium and PTH. In a run-in period of 7 days prior to the study, regular treatment with phosphate and calcitriol was discontinued. During the subsequent 48-hour study period the patients received phosphate supplement. Alon et al. [14] demonstrated that a single dose of cinacalcet (30 or 60 mg, depending on weight) on day two reduced PTH and plasma calcium significantly compared to baseline values.

We measured equally low levels of calcium on only one occasion, when our patient was treated with a daily dose of 60 mg cinacalcet (October 2010 as shown in Figure 1). It seems reasonable to assume that treatment with activated vitamin D analogues as in our case but which is absent in the study by Alon et al. [14] will tend to attenuate this calcium lowering effect of cinacalcet.

### 3.2. Effect on Plasma Phosphate

Following the reduction of plasma PTH and hence reduced renal phosphate loss, an increase in plasma phosphate may be expected. In a study including two patients with FGF-23 mediated tumor induced osteomalacia, adjuvant therapy with cinacalcet led to increased plasma phosphate levels and allowed a substantial reduction of calcitriol and phosphate supplements [17]. A similar phosphate-saving effect of cinacalcet has been suggested in patients with XLH [14].

However, plasma phosphate remained stable in our patient despite reduced plasma PTH levels and unchanged phosphate supplements. In the case series by Yavropoulou et al. [16], plasma phosphate as well as TmP/GFR increased. Interestingly, this occurred with a concomitant normalization of FGF-23, which most likely contributed to the observed increase in plasma phosphate levels. However, in the two patients with FGF-23 mediated tumor induced osteomalacia studied by Geller et al. [17], the increase in plasma phosphate and TmP/GFR occurred despite increased FGF-23 levels during treatment with cinacalcet. Unfortunately, we were not able to measure FGF-23 before or during treatment with cinacalcet. Further studies are needed to assess possible effects of cinacalcet on plasma phosphate and FGF-23, including whether an effect may vary according to the etiology of hypophosphatemia and between different genetic mutations in patients with HR.

### 3.3. Effect on ALP

Despite decreased PTH levels, we observed increased levels of ALP during cinacalcet treatment. We speculate that this may be a consequence of treatment with cinacalcet. Our patient had a stable calcidiol level and she did not suffer any fractures. Her orthopedic operation was performed 2.5 years after initiation of cinacalcet treatment. The levels of ALP were not reported in any of the previously mentioned studies of XLH patients. Reports on changes in levels of ALP during treatment with calcimimetic agents are conflicting. Several studies have established that treating SHPT in hemodialysis patients with calcimimetic agents results in a decrease in ALP [18]. However, in an open labeled study of mild PHPT approached with cinacalcet during a period of 5 years, increased ALP levels were reported [15]. Dvorak et al. [19] have demonstrated that both high levels of calcium and the CaSR agonist gadolinium stimulate signaling associated with proliferation in osteoblasts and upregulate mRNA responsible for osteoblast differentiation and mineralization. Thus, a rise in ALP could be a marker of improved bone formation induced by cinacalcet. However, bone mineral density has not been shown to increase in response to treatment with cinacalcet in patients with PHPT, suggesting that a potential bone anabolic effect is of minor importance [15]. Our findings need to be confirmed by others, and the pathophysiological mechanism should be further elucidated.

## 4. Conclusion

Long term treatment with cinacalcet for a period of three years showed sustained dose-dependent response on PTH levels, and cinacalcet appeared to be safe and well tolerated. Cinacalcet may therefore be a beneficial therapeutic option in XLH with SHPT. However, we recommend close control of plasma calcium to detect hypocalcemia which seems to be a potential adverse effect of treatment. A possible effect on plasma phosphate and alkaline phosphatase levels needs further investigations. Randomized clinical studies should be performed before cinacalcet can be recommended as standard treatment in XLH complicated by SHPT.

## Figures and Tables

**Table 1:** Effect of cinacalcet treatment on biochemical indices of calcium homeostasis in a patient with X-linked dominant hypophosphatemia.

Plasma (reference interval)	Before cinacalcet treatment	During cinacalcet treatment	*P* value
PTH, pmol/L (1.6–6.9)	9.3 ± 3.3	5.4 ± 3.2	0.03
Calcium-ionized, mmol/L (1.18–1.32)	1.25 ± 0.03	1.15 ± 0.04	<0.01
Phosphate, mmol/L (0.76–1.41)	0.84 ± 0.10	0.80 ± 0.08	0.46
Alkaline phosphatase U/L (35–105)	94 ± 7	118 ± 8	<0.001
Calcidiol, nmol/L (50–160)	72 ± 20	72 ± 8	0.96
Creatinine, *μ*mol/L (45–90)	58 ± 2	61 ± 4	0.17

Mean plasma levels (±standard deviation) in the three years prior to the start of treatment and during three years of treatment with cinacalcet. *P* values denote difference in mean values.
